# Novel dual convolution adaptive focus neural network for book genre classification

**DOI:** 10.1371/journal.pone.0331011

**Published:** 2025-11-07

**Authors:** Qingtao Zeng, Lixin Zhang, Jiefeng Zhao, Anping Xu, Yali Qi, Liqin Yu, Wenjing Li, Haochang Xia

**Affiliations:** 1 Beijing Institute of Graphic Communication, Beijing, China; 2 College of Acupuncture-Moxibustion and Tuina, Beijing University of Chinese Medicine, Beijing, China; 3 State Key Laboratory of Networking and Switching Technology (Beijing University of Posts and Telecommunications), Beijing, China; 4 Zhejiang Institute of Mechanical and Electrical Engineering Co.Ltd, Hangzhou, China; University of Lagos Faculty of Engineering, NIGERIA

## Abstract

Book covers typically contain a wealth of information. With the annual increase in the number of books published, deep learning has been utilised to achieve automatic identification and classification of book covers. This approach overcomes the inefficiency of traditional manual classification operations and enhances the management efficiency of modern book retrieval systems. In the realm of computer vision, the YOLO algorithm has garnered significant attention owing to its excellent performance across various visual tasks. Therefore, this study introduces the CPPDE-YOLO model, a novel dual-convolution adaptive focus neural network that integrates the PConv and PWConv operators, alongside dynamic sampling technology and efficient multi-scale attention. By incorporating specific enhancement features, the original YOLOv8 framework has been optimised to yield superior performance in book cover classification. The aim of this model is to significantly enhance the accuracy of image classification by refining the algorithm. For effective book cover classification, it is imperative to consider complex global feature information to capture intricate features while managing computational costs. To address this, we propose a hybrid model that integrates parallel convolution and point-by-point convolution within the backbone network, integrating it into the DualConv framework to capture complex feature information. Moreover, we integrate the efficient multi-scale attention mechanism into each cross stage partial network fusion residual block in the head section to focus on learning key features for more precise classification. The dynamic sampling method is employed instead of the traditional UPsample method to overcome its inherent limitations. Finally, experimental results on real datasets validate the performance enhancement of our proposed CPPDE-YOLO network structure compared to the original YOLOv8 classification structure, achieving *Top_1 Accuracy* and *Top_5 Accuracy* improvement of 1.1% and 1.0%, respectively. This underscores the effectiveness of our proposed algorithm in enhancing book genre classification.

## Introduction

With the advancement in printing technology and the growth of various industries, individuals in the information age are presented with a diverse array of book genres to explore. In the fast-paced contemporary lifestyle, readers often seek to ascertain whether a book’s genre aligns with their interests prior to delving into its contents. Therefore, categorising book genres and creating a genre bibliography can assist readers in selecting books that resonate with their preferences and streamline their decision-making process. However, categorising genres for both e-books and traditional paper books is a complex and time-consuming task. In the era of big data, leveraging deep learning algorithms becomes imperative and pivotal in achieving book genre classification. Facilitating swift and efficient differentiation of book types and establishing genre reference bibliographies not only aids readers in promptly identifying books of interest but also fosters the rapid development of the book industry to a certain extent.

Image classification stands as a critical task within computer vision, drawing inspiration from the human visual system. Its primary objective lies in identifying and categorising objects or scenes depicted in input images, facilitated by algorithms that analyse the visual attributes of the imagery. Target classification techniques find increasing applications across diverse domains such as medical imaging analysis, autonomous driving obstacle detection, pest and disease identification, crop species monitoring in agriculture, and environmental preservation via satellite or drone image classification. These techniques underscore automation and intelligence, contributing significantly to various fields. Notably, recent years have witnessed significant advancements in image classification, propelled by the development of deep learning techniques, resulting in the development of more accurate and dependable algorithms tailored for practical applications.

In this paper, we introduce the novel CPPDE-YOLO, a dual convolutional adaptive focusing neural network. The primary contributions of this study are as follows.

In order to improve the classification accuracy, enhance the feature extraction ability of the model, and improve the performance of the model in dealing with complex datasets, we propose a hybrid model based on PConv and PWConv, and incorporate the core concepts of DualConv to further effectively capture the complex feature information. In order to improve the focusing ability of key features, we incorporate an effective multi-scale attention mechanism in the network fusion residual block of each cross-stage part of the head network part, and employ the DySample technique to overcome the limitations inherent in the UpSample approach and enhance the flexibility of the network in dealing with features at different scales.

Through experiments conducted on datasets, particularly focusing on image classification tasks, we validated the superior classification efficiency and accuracy of the CPPDE-YOLO network in comparison to the original YOLOv8 model.

The remainder of the paper is organised as follows. we first review related work on image classification. We then present a detailed description of the proposed CPPDE-YOLO algorithm structure. Subsequently, we analyse the experimental results. Finally, we conclude the paper and suggest directions for future research.

## Related work

Over time, image classification algorithms have progressed from simple techniques to the current utilisation of deep learning for designing network architectures. There has been a concerted effort towards enhancing classification accuracy and optimising network structures.

The application of the K-Nearest Neighbours (KNN) concept in deep learning can be traced back to the 1950s [1,2]. Additionally, statistician and mathematician R. A. Fisher pioneered the concept of nearest-neighbor classification [3,4], which serves as a precursor to the K-NN algorithm. Fisher’s contributions significantly influenced the field of pattern recognition. In a seminal paper, T. Cover and P. Hart formally proposed the K-NN algorithm. Thomas Cover, in his later research, conducted an in-depth study of the K-NN algorithm and proposed some improvements, such as approximate nearest neighbour search, which speeds up the computation process and improves the efficiency of the algorithm by reducing the search complexity [5].

Convolutional neural networks(CNN) [6,7] are primary algorithms employed in computer vision and were initially proposed in the 1990s. Early research on neural networks laid the groundwork for the development of CNN. LeNet-5, an early form of CNN proposed by Y. LeCun et al., served as a pivotal starting point for CNN. The emergence of AlexNet [8] sparked a renaissance in deep learning, leading to the widespread adoption of CNN. VGGNet [9,10] improved performance by employing small convolutional kernels and a deep network architecture. GoogLeNet [11] addressed computational complexity by introducing an inception module, whereas ResNet architecture handled it by introducing residual connections, mitigating the gradient vanishing problem and enabling the training of deeper layers [12]. CNN algorithms excel in processing image data [13], learning spatial hierarchical features through multi-layered networks that alternate between convolutional, pooled, and fully connected layers.

The transformer model [14], initially designed for natural language processing, can also be adapted for image classification tasks through the integration of a self-attention mechanism, as demonstrated in Equation (1).


$$Attention(Q,K,V)=softmax\left(\frac{QK^{T}}{\sqrt{d_{k}}}\right)V$$ (1)

The query, key, and value matrices are denoted by Q,K,V respectively. The dimensional representation of the key is denoted by $d_{k}$.

The You Only Look Once (YOLO) family of algorithms [15] constitutes a CNN-based evolutionary network capable of performing various computer vision tasks, including target detection and classification. In 2015, YOLOv1 was introduced, and Joseph Redmon and others innovated by proposing a one-step approach to the detection task.

In 2016, YOLOv2 introduced the concept of ‘anchor points’, which improved the accuracy and computational speed of the model. YOLOv3, released in 2018, further enhanced the model’s accuracy and introduced the innovative idea of residual connectivity. Subsequent versions such as YOLOv4 and YOLOv5, proposed in 2020, optimised the accuracy and computational speed of the network. In 2021, YOLOv6 was updated; it proposed a network based on the RepVGG skeleton [16], which exhibits greater parallelism than previous versions. YOLOv7, released in 2022, featured improvements in terms of reduced parameter count, computational effort, and enhanced accuracy. In 2023, Ultralytics released the YOLOv8 model, which outperforms previous YOLO algorithms in the same family and is compatible and scalable with all current versions of the YOLO algorithm.

Our study aimed to classify book covers using the YOLOv8 algorithm. We enhanced this algorithm by introducing a hybrid model that integrates parallel convolution and point-by-point convolution. Additionally, we introduced the expectation Efficient Multi-Scale Attention mechanism into each C2f residual block in the head section. Meanwhile, the dynamic sampling method is employed instead of the traditional Upsample method to address its limitations. A comprehensive series of experiments were conducted to derive a classification model suited to our requirements.

## Model

Prior to presenting our proposed model, we will outline the YOLOv8 and FasterNet backbone architectures.

**Fig 1:**
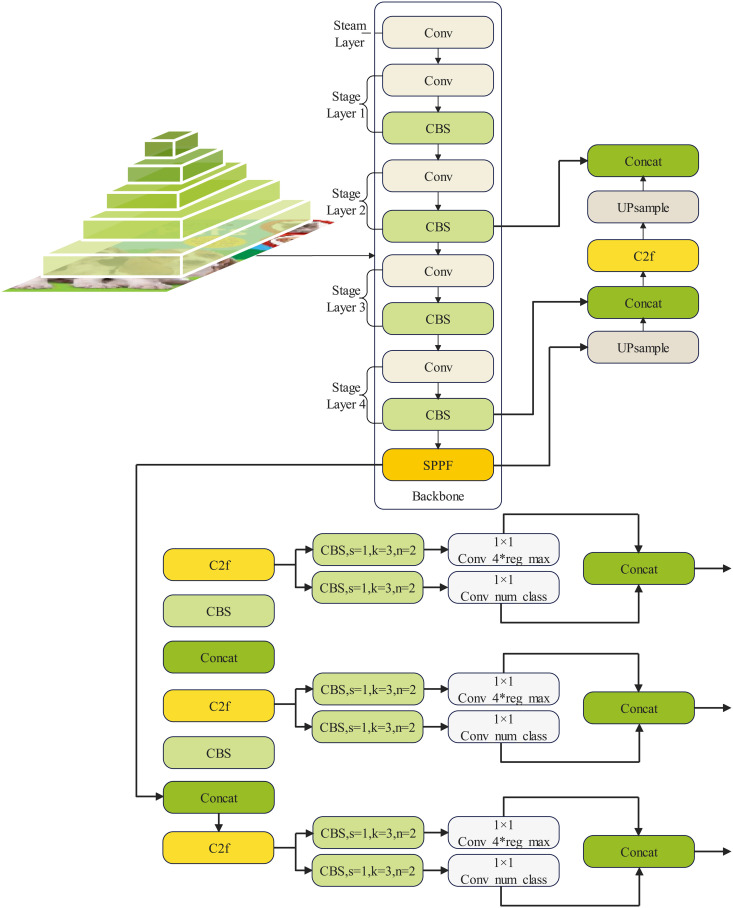
Structure of the YOLOv8 network.

The YOLOv8 algorithm, introduced by Ultralytics, represents the latest advancement in the YOLO series, designed for tasks such as image classification, target detection, and instance segmentation. In comparison to the YOLOv5 algorithm, also released by Ultralytics, YOLOv8 has improved speed and accuracy. Fig 1 illustrates the architecture of YOLOv8.

Fig 1 demonstrates the evolutionary improvement embodied in the YOLOv8 algorithm over its predecessors in the YOLO series. The C2f module replaces the C3 module in the YOLOv5 structure, resulting in a lighter network design.

**Fig 2:**
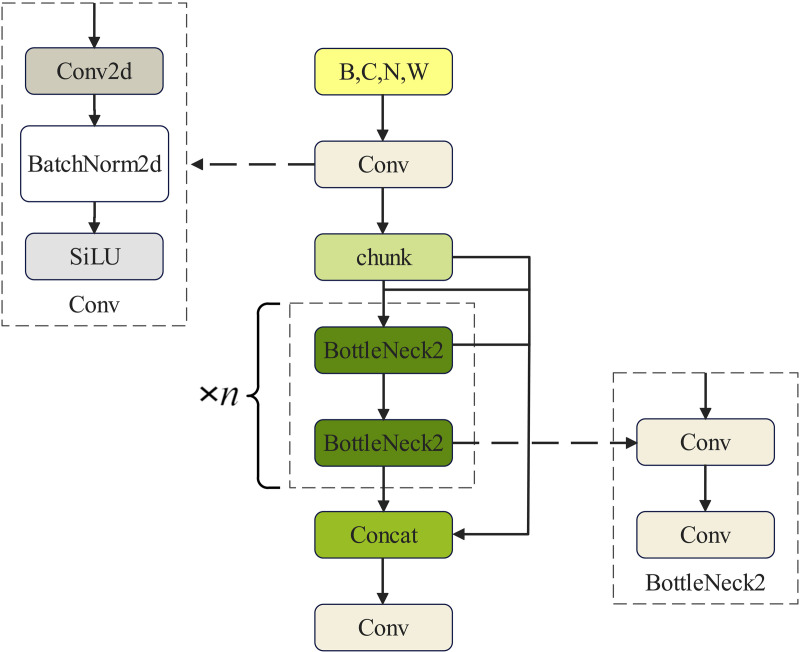
Schematic diagram of the structure of the C2f section in YOLOv8.

Fig 2 provides an insight into the modified C2f module, showcasing additional jump-layer connections and the elimination of the convolution operation in the original branch. The primary function of the C2f module is to facilitate effective feature extraction for targets of varying sizes. This is accomplished through the construction of a pyramid structure featuring different pooling layers within the module. Subsequently, information exchange between feature maps at different levels occurs, effectively broadening the perceptual range of the network model and enhancing the accuracy of the relevant task.

In Fig 2, C,H and W represent the number of channels, height, and width of the input feature image, respectively. B denotes the batch size, while the term ‘chunk part’ denotes the division of corresponding feature maps into a specific number of chunks across corresponding dimensions.

YOLOv8 [17] introduced a novel loss function, utilising VFL-Loss as the classification loss, which represents an enhanced version of the cross-entropy loss. The cross-entropy loss, also referred to as the logarithmic loss, is a widely adopted loss function in classification tasks.

The experimental task that we are working on involves a multi-classification task, which is an extension of a binary classification task. Equations (2) and (3) illustrate the relationship between the two tasks and encapsulate the core idea.


$$\begin{array}{c} @cP=\frac{\text{1}}{Y}\sum_{i}-\left[n_{i}\cdot log\left(Q_{i}\right)+\left(\text{1}-n_{i}\right)\cdot log\left(\text{1}-Q_{i}\right)\right]\\ \left\{ \;\;\;\begin{array}{c} n_{i}\Rightarrow Label\;of\;sample\;i\left\{ \begin{array}{c} @l\text{1},positive\;class\\ \text{0},negative\;class \end{array}\right.\;\\ Q_{i}\Rightarrow Probability\;that\;sample\;i\;is\;predicted\;to\;be\;a\;positive\;class \end{array}\right.\; \end{array}$$ (2)

(P=1Y∑iLi=−1Y∑i∑c=1Myiclog(pic){@lM⇒Number of categoriesyic⟹{@l0,if the true category of sample i is equal to c1,if not pic⟹Predicted probability that observation sample i belongs to category c ) (3)

The primary modification in YOLOv8 entails the adoption of the decoupled head structure within the head section. This partition separates the classification and detection heads, thereby enhancing flexibility in adjusting relevant parameters and facilitating seamless migration and adaptation across different tasks. The specialisation of each head for its respective task holds the potential to enhance overall task performance. Consequently, we opted for YOLOv8 as the initial model to train the network, considering it as a prototype.

The Fasternet network [18] presents a neural network structure engineered to enhance efficiency in floating-point computation. This design reduces network latency by innovatively and specifically crafting modules to accelerate model computation across diverse hardware devices while preserving accuracy. The Fasternet network [19,20] introduces the PConv operator, which adeptly extracts spatial features while curtailing unnecessary computations and memory accesses inherent in traditional convolution. Equation 4 elucidates the operation flow of the PConv operator.


$$H\times W\times k^{\text{2}}\times c_{p}^{\text{2}}$$ (4)

**Fig 3:**
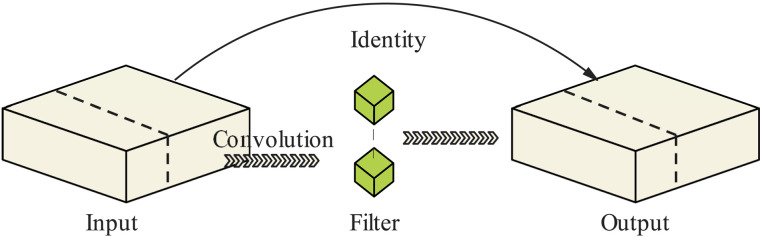
Concise sample of the core concept of PConv.

In Equation (4), H and W denote the height and width of the input feature map, respectively. k denotes the size of the convolution kernel, c denotes the number of channels of the output feature map, and $c_{p}$ denotes the number of channels used for spatial feature extraction by PConv in the operation. The equation demonstrates that reducing the amount of computation leads to faster computational speed for the network. The introduction of the PConv layer aims to diminish computation and eliminate redundancy while processing sufficient information, thereby enhancing the network’s speed and computational efficiency. The structure of the PConv layer is depicted in Fig 3.

**Fig 4:**
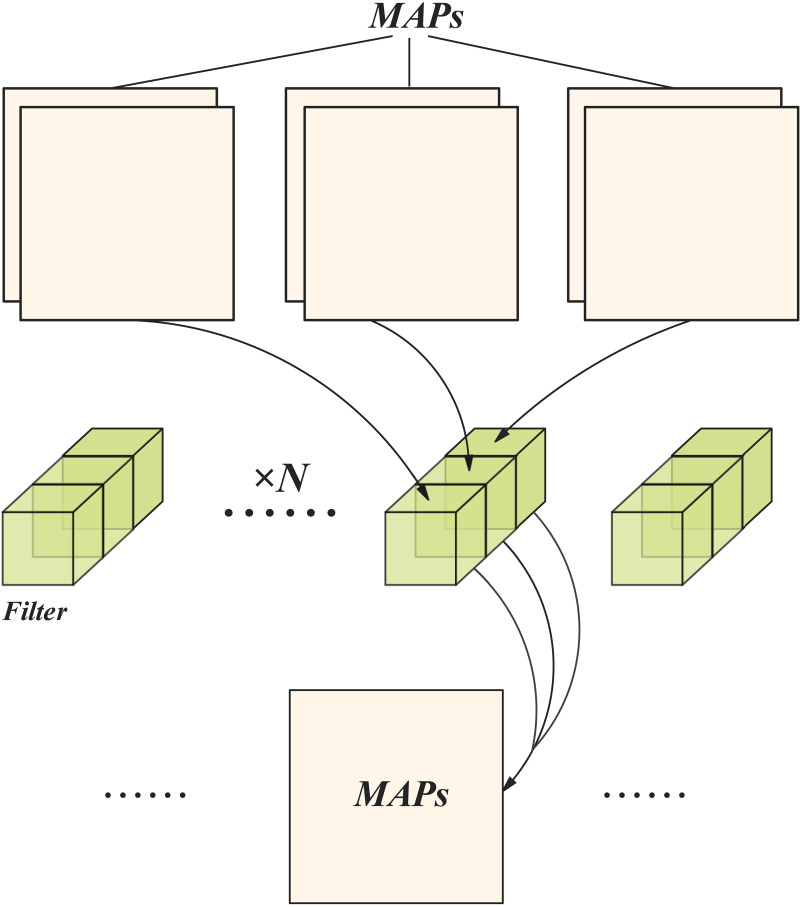
Concise sample of the core concept behind PWConv.


In a point-by-point convolutional layer, for each input feature map, c computations are performed to generate a channel of the output feature map. This enables independent updating of each channel’s information without altering the size of the input feature map. Fig 4 illustrates the pathway structure of the PWConv operator [21]. Equation (5) delineates the computational flow of point-by-point convolution (PWConv). H,W denote the height and width of the input feature map, respectively, and H represents the number of channels of the output feature map.

$$H\times W\times c^{\text{2}}$$ (5)

$$H\times W\times \left(k^{\text{2}}\times c_{p}^{\text{2}}+c^{\text{2}}\right)$$ (6)

Fasternet streamlines the network structure by incorporating PConv and PWConv operators in the middle of the network. Equation (6) represents the traffic calculation formula for PConv and PWConv, enabling high performance across a variety of hardware devices.

The network’s performance can be significantly enhanced by leveraging a combined model of partial and point-by-point convolution. Point-by-point convolution facilitates the transfer of information between channels while preserving spatial dimensions. Moreover, a combination of PConv and PWConv can be employed, where PConv employs reduced channel arithmetic and PWConv facilitates comprehensive integration and updating of information between channels. Through the utilisation of both modules, the network model becomes inherently more flexible and expandable.

### Algorithm

**Fig 5:**
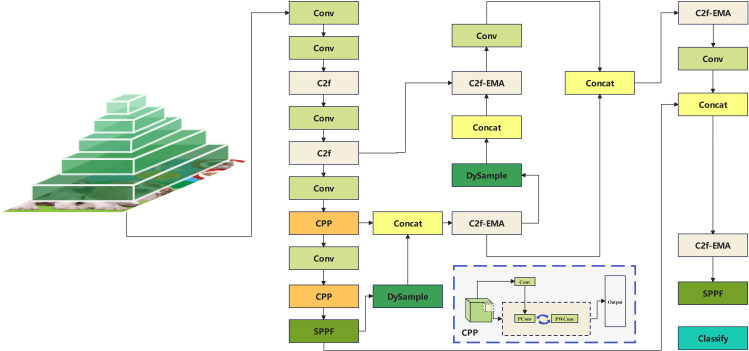
Schematic of the CPPDE-YOLO network structure.

Building upon the framework of the YOLOv8 algorithm, we amalgamated the features pertinent to image multi-classification tasks to enhance the classification network tailored for book datasets. Our innovative contribution is the CPPDE-YOLO network structure, depicted in Fig 5.

The fundamental concept of the Fasternet network was integrated into the backbone network of YOLOv8 by introducing integrated PConv and PWConv. Through the incorporation of integrated PConv and PWConv within the DualConv framework, certain conventional convolutional layers in the original network were replaced. This leads to optimised information processing and feature extraction, while also managing computational costs and parameter counts effectively.

Integrated PConv contributes to only 6.25% of the traffic compared to conventional convolution, thereby lightening the network structure to some extent. PConv selectively performs the Conv operation on a subset of input channels to extract spatial features, while the remaining channels are reserved for subsequent pointwise convolution operations.


$$\begin{array}{c} Y_{h,w,k}=\sum_{n=\text{1}}^{N}X_{h,w,n}\cdot W_{k,n}+B_{k} \end{array}$$ (7)

PWConv is also referred to as a 1×1 convolution, as illustrated in Equation (7), where Y is the output feature map and X is the input channels, i.e., each spatial location (h,w) independently performs a linear combination between channels. $W_{k,n}$ denotes the representation of the weight of the kth output channel to the nth input channel, and $B_{k}$ denotes the bias term. This function primarily serves to adjust the number of channels between each network layer, and its utilisation enables the reorganisation and integration of feature information without altering the spatial dimensions of the feature map through linear transformation.

DualConv combines both 3×3 and 1×1 convolutional kernels to simultaneously process the same input feature channels. One convolutional kernel aids the network in extracting more spatial information during the feature extraction phase, whereas the other facilitates the interaction and integration of feature channels under controlled computational complexity and parameter counts, leveraging the concept of group convolution. Group convolution optimises the arrangement of convolutional filters by amalgamating network properties, thus reducing model complexity and enhancing information processing efficiency.

**Fig 6:**
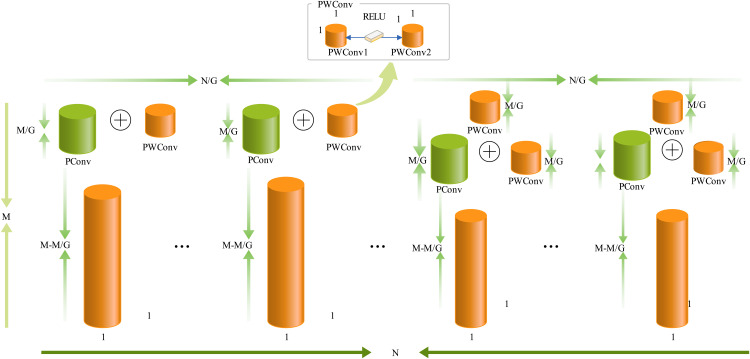
Schematic structure of the improved DualP_PWConv.

The central idea behind the DualConv structure [22] is to capitalise on the strengths of both convolutional kernels. As depicted in Fig 6, we aim to stack a PWConv atop a PConv. This configuration ensures that the feature information retained after a partial convolution computation can be effectively processed by a subsequent PWConv, guaranteeing comprehensive capture of information across all channels. Leveraging DualConv as a framework, PConv and PWConv are introduced, and an activation function is applied to construct a novel network architecture.

**Fig 7:**
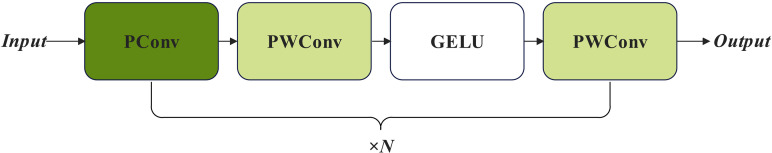
Computational flow of the PConv and PWConv parts within the improved structure.

As depicted in Fig 7, the replacement of the original convolutional kernels in DualConv with PConv and PWConv results in a more centrally focused network configuration compared with the original conventional conv layer in DualConv. This enhancement maximises network performance and enables more accurate execution of image classification tasks.

Given the intricate categories and complex features of the dataset, we streamlined the experimental network structure to explore a rational configuration. Instead of utilising the classification header of YOLOv8 itself, we emulated the structure of the detection header in our experiments, preserving the up-sampling and fully connected processing scheme. Additionally, we proposed the introduction of a dynamic up-sampling structure in lieu of the original up-sampling structure to operate within the head section. Moreover, we explored the integration of the EMA mechanism within the head section at the location of the multi-layered C2f module [23].

DySample [24] represents a dynamic up-sampling method that differs from traditional up-sampling approaches. In contrast to conventional methods that adjust the feature map by generating a static convolution kernel, DySample dynamically adjusts sampling points based on image content interpolation. This approach aims to recover detailed feature information without introducing redundancy. As shown in Equation (8), Y is the sampling output, I(X) is the interpolated feature map, F(X) is the linear connectivity layer, S is the dynamic range factor, G is the initial grid sampling. DySample ensures uniform distribution of sampling points across the feature map by adjusting the scaling factor of the offset. This technique minimises overlapping and blank areas in the feature image, thereby ensuring richness and completeness of feature information during the up-sampling process.


$$Y={gird}_{\_}sample\left(I(X),\left(G+F(X)\cdot S\right)\right)$$ (8)

DySample primarily targets dense prediction tasks; we innovatively introduced it to the image classification task to optimise the traditional up-sampling process. By integrating DySample and leveraging the fully connected layer, we aim to generate a stable output and enrich the information provided.

The EMA attention mechanism adopts a hybrid cross-channel and spatial information approach, and the EMA attention mechanism contains parallel submodules, which can effectively help the model to capture cross-dimensional feature maps, realise cross-dimensional information interactions, and establish dependencies between different dimensions. The structure of the EMA attention mechanism includes two output branches, one is a 3×3 branch and the other is a 1×1 branch, and then the global spatial information in the output of the 1×1 branch is encoded by using two-dimensional global average pooling, and its operation principle formula is shown in Equation (9).


$$\begin{array}{c} Z_{c}=\frac{\text{1}}{H\times W}\sum_{j}^{H}\sum_{i}^{W}X_{c}(i,j) \end{array}$$ (9)

**Fig 8:**
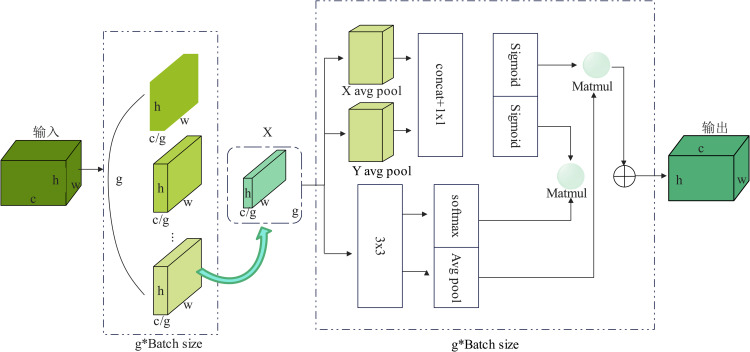
Example of the EMA attention mechanism structure.

The structure of the EMA attention mechanism is shown in Fig 8, which enables the model to dynamically adjust its attention regions for the input feature map, choosing to focus on the feature region that is most important for the current task execution, and improving the accuracy and robustness of the model’s detection of the feature points under complex backgrounds, and selecting the EMA attention mechanism to be inserted as the attention mechanism in the C2f residual block can be effectively combined with the dynamic upsampling can be effectively combined to improve the performance of the network model to a greater extent, and can also reduce the interference information caused by redundant noise to a certain extent.

Our proposed CPPDE-YOLO structure, to a certain extent, replicates the high compactness of the original YOLOv8 structure and demonstrates robust performance in the final image classification task [25,26] through the synergistic integration of multiple modules. The following pseudo-code (Table 1) highlights the key aspects of the CPPDE-YOLO network construction [27] and provides a general overview of how the proposed modules collaboratively contribute to improving the performance and efficiency of the model.

**Table 1:** Pseudocode of the CPPDE-YOLO Model Workflow.

**input:** An input tensor *X* of size *C* × *H* × *W* **output:** A processed feature tensor *Y* **Function** DualConv (*X*, *C*_*in*_, *C*_*out*_, *kernel*_*size*_, *stride,padding*); mask←Mask Generation Convolution(X, $C_{in}$ , $C_{out}$ /4); *groupedFeature*← GroupedConvolution(*mask*, *C*_*in*_, *C*_*out*_, *kernel*_*size*_, *stride, padding);* *processedFeature*← PointwiseConvolution(*groupedFeature*, *C*_*out*_, *C*_*out*_); **return** processedFeature; end **Function** Forward(*X*): dualFeature ←*DualConv*(*X*, *C*_*in*_, *C*_*out*_, *kernel*_*size*_, *stride, padding*); upsampledFeature ← DynamicUpsampling(dualFeature); *Y* ← AttentionMechanism(*upsampledFeature*,*C*_*out*_); **return** *Y*; end

## Experiments

The experimental platform comprised a six-core Intel Core i7 processor and an NVIDIA GeForce RTX 4090 graphics card. All experiments were implemented using the PyTorch framework (Python 3.8.18). The learning rate was set to 0.01, with 200 training epochs and a momentum of 0.937, and SGD was employed as the optimizer. Mixup was consistently applied as the data augmentation technique throughout the experiments.

The data used for the experiment came from the Book Cover Dataset [28]. All book cover images are hosted by and copyright Amazon.com, Inc. The use of the book cover images is fair use for academic purposes. The dataset contains 57,000 book cover images across 30 categories and was divided into 80% for training and 20% for testing.

We selected *Top_1 Accuracy* and *Top_5 Accuracy* as the experimental general evaluation indices. *Top_1 Accuracy* indicates that the prediction of the first category corresponds to the accuracy of the actual results, and *Top_5 Accuracy* indicates the accuracy of the predictions of the first five categories containing the actual results.

Given that our dataset primarily comprise feature images of book covers for book type classification, heavily reliant on input features, our exploration of network models for this classification task emphasised enhancing the efficiency of utilising input feature information alongside classification accuracy. Table 2 documents the performance of various network model structures on the dataset observed during the exploration process.

**Table 2:** Accuracy performance of four network structures.

Network Structure	*Top_1 Accuracy/%*	*Top_5 Accuracy/%*
① Ghost-yolo	27.4	59.3
② Faster-yolo	28.0	59.8
③ DualPConv-yolo	27.8	59.9
④ DualP_PWconv-yolo	28.5	60.0

In the Backbone section, we explored several model configurations. Model ① leverages Huawei’s mobile model, Ghostnetv2. Considering the prevalent use of handheld mobile devices for book classification tasks, this model was considered. Model ② attempts to integrate the FasterNeT network into the YOLOv8 backbone but exhibits lower accuracy due to the simplified network structure.

**Fig 9:**
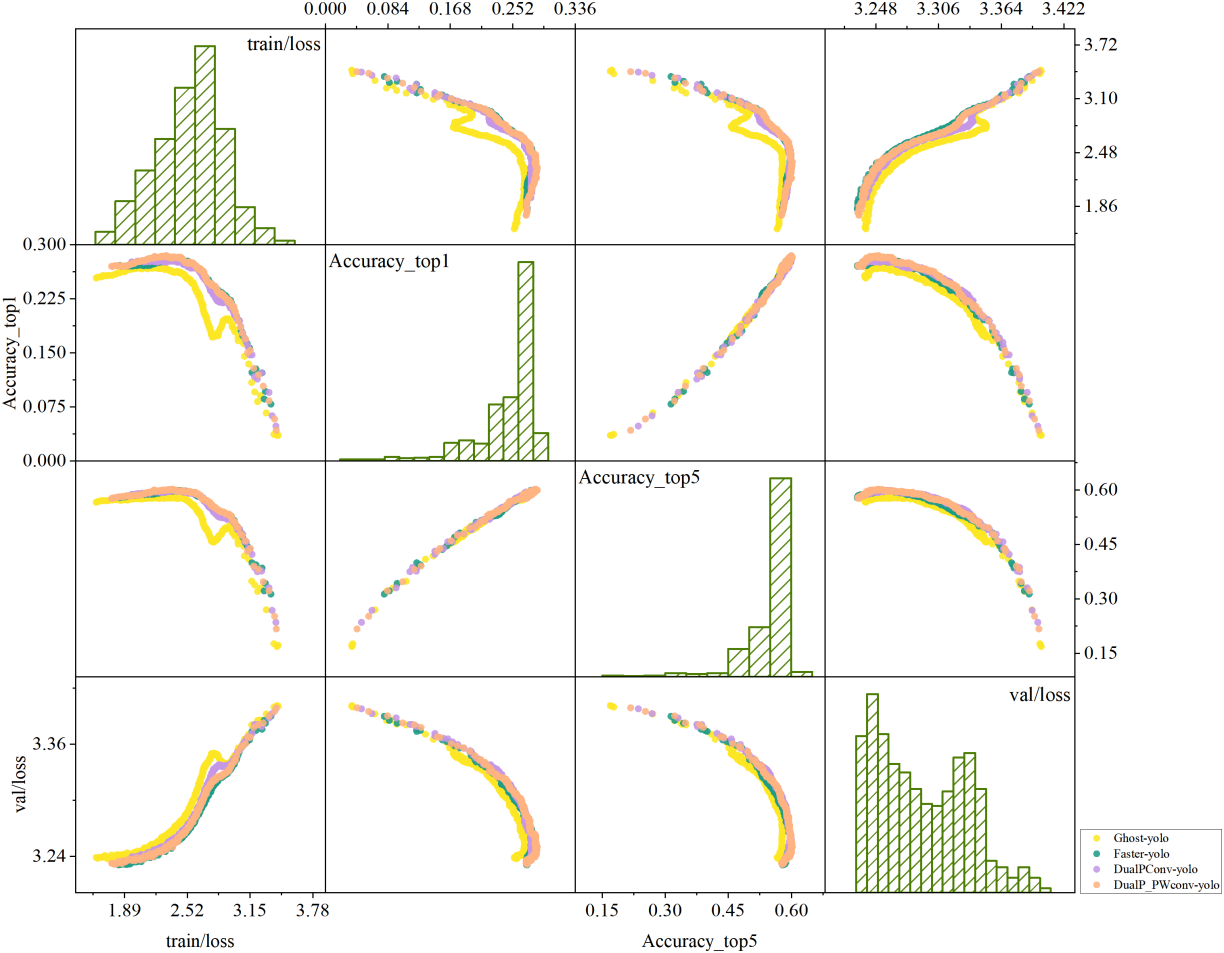
Correlation Matrix of Loss and Accuracy between Training and Validation for Different Models.

Models ③ and ④ both adopt the DualConv concept by replacing the original network’s convolution kernel with either a PConv superposition or a combination of PConv and PWConv superposition, respectively, intending to partially substitute the residual block network in the YOLOv8 backbone. As depicted in Fig 9, the experiments demonstrate that the network’s accuracy improves by 0.7% for *Top_1 Accuracy* and 0.1% for *Top_5 Accuracy* when using both PConv and PWConv compared to the network with only the PConv operator.

In the aforementioned experiments, we observed that the network configuration following the PWConv operator outperformed that following the PConv. Consequently, we conducted comparative experiments regarding the number of PConv and PWConv operators and their configuration, as detailed in Table 3.

**Table 3:** Possible configuration schemes for multiple PConv and PWConv.

Network Structure	*Top_1 Accuracy/%*	*Top_5 Accuracy/%*
①DualPConv*4-yolo	27.8	59.9
②DualPConv*3-yolo	28.5	60.0
③DualP_PWConv*3-yolo	28.6	60.1
④DualP_PWConv*3_EMA-yolo	28.5	59.7
⑤DualP_PWConv*2-yolo	28.8	60.6

Table 3 illustrates the enhancements made to the backbone section of the base network in YOLOv8 when experimenting with different configurations of the DualP_PWConv layer and the EMA attention mechanism. It presents the performance of various network architectures in terms of accuracy. The numbers following DualP_PWConv indicate the quantity of such pairings employed in the backbone.

The experimental outcomes reveal that structure ①, utilising PConv alone as a replacement for the original convolutional kernel of DualConv, is less effective compared to replacing the original convolutional kernel with PConv and PWConv operators. Under similar conditions, the *Top_1 Accuracy* of structure ③ increases by 0.1% compared to ②, and the *Top_5 Accuracy/*increases by 0.1% compared to ②, highlighting the beneficial role of the PWConv layer in enhancing network performance.The experimental results from groups ③ and ④ indicate that the EMA attention mechanism does not consistently improve performance under certain configurations. Placing the EMA attention mechanism in the backbone network does not always optimise network performance to its full potential.

**Fig 10:**
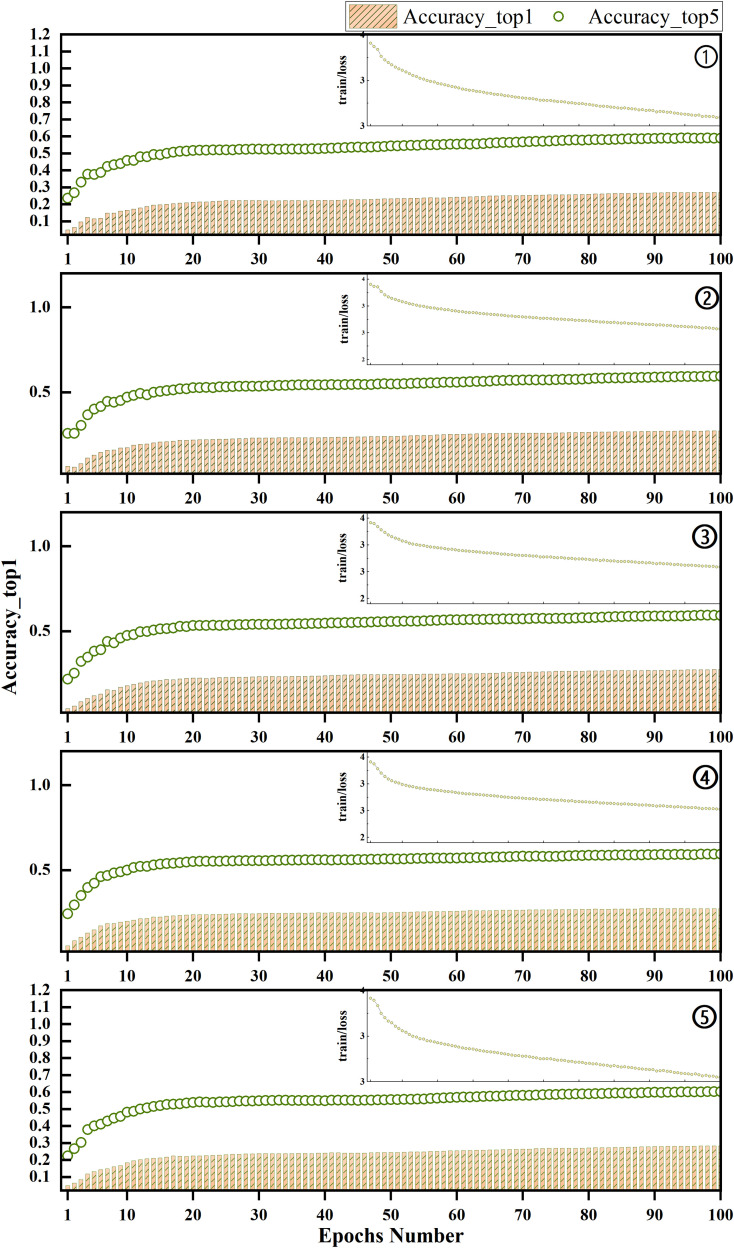
Evolution of training loss and accuracy for the five network configurations in Table 3.

In the DualP_PWConv layer pairing experiment, three configuration scenarios were explored for the backbone network part. It was observed that reducing the number of layers from three in ③ to two in ⑤ led to successive performance improvements, *Top_1 Accuracy* with a difference of 0.2% before and after. Additionally, Fig 10 confirms that reducing the number of layers within a certain range does not detrimentally impact performance; rather, it reduces model complexity, thereby enhancing performance to some extent.

Based on the aforementioned experiments, we determined that the DualP_PWConv network is effective, while the EMA attention mechanism does not yield significant advantages in the backbone. Consequently, we proceeded to investigate the layer scheme of the EMA attention mechanism and also attempted to incorporate the DySample sampling technique in the head section of the network.

Table 4 shows different combinations of modules within the network layer. Table 4 illustrates that adjusting the number of DualP_PWConv within a specific range tends to enhance the efficiency and accuracy of the model. As observed from configurations ① to ⑤, reducing the complexity in this setup generally leads to improved network performance.

**Table 4:** Ablation Study on the Number of Modules Introduced in the Network Layer.

NO.	DualP_PWConv	UpSample	DySample	EMA	*Top_1 Accuracy/%*	*Top_5 Accuracy/%*
①	4	–	2	4	29.8	61.3
②	3	2	–	4	29.9	62.2
③	3	–	2	3	29.9	61.8
④	3	–	2	4	30.3	61.6
⑤	2	–	2	4	30.7	62.7

The integration of dynamic sampling into the model demonstrates a noticeable improvement in performance. For instance, configuration ② utilises up-sampling instead of dynamic sampling, resulting in slightly inferior performance compared to other network structures employing dynamic sampling. This indicates that DySample is particularly effective when resizing feature maps, preserving detailed information more effectively.

A comparison between configurations ③ and ④ reveals that increasing the number of EMA attention mechanisms within the same setup enhances network performance. This underscores the effectiveness of the attention mechanism in enhancing the model’s capability to capture crucial features.

**Fig 11:**
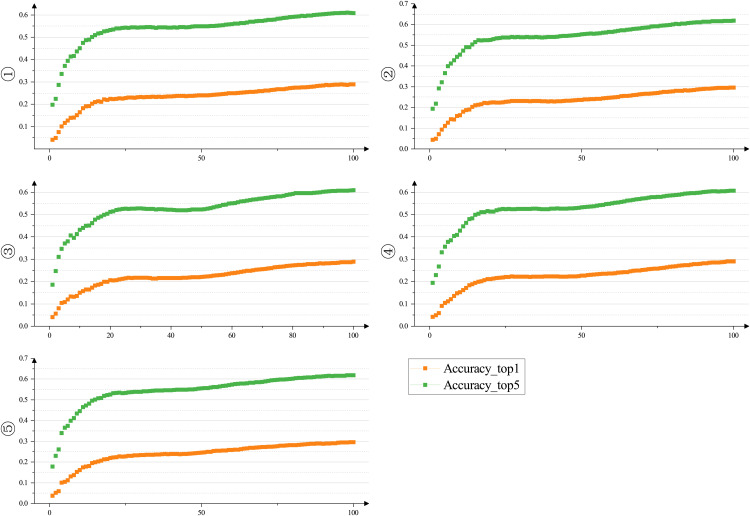
Trend of accuracy for the five network configurations in Table 4.

As depicted in Fig 11 above, Scheme ⑤ stands out as the top-performing configuration among all network structures, representing the architecture of our proposed CPPDE-YOLO network model. Through the incorporation of dynamic sampling and the EMA attention mechanism, along with the judicious design of the DualP_PWConv layer, the classification accuracy and overall performance of the model can be effectively enhanced to a considerable extent.

Table 5 presents the detailed classification accuracy of the CPPDE-YOLO network structure across 30 categories when classifying book genres. Notably, the *Top_1 Accuracy* and *Top_5 Accuracy* of the Comics & Graphic Novels category is remarkable, with 65.62% for the top prediction and 86.88% for the top five predictions, both of which are the highest accuracies in the table. Moreover, the Calendars category demonstrates exceptionally high classification accuracy, particularly in the first five predictions, all of which are identified with high precision. Similarly, the Test Preparation category exhibits commendable accuracy, with *Top_5 Accuracy* reaching 74.37%, indicating distinctive feature maps easily discerned by the network. Conversely, the Humour & Entertainment category shows lower recognition accuracy, likely due to the subjective nature of its content style and weak association with key subject words, resulting in reduced classification accuracy. The *Top_1 Accuracy* for Health, Fitness & Dieting category registers the lowest accuracy at only 7.20%, with the top five predictions at 44.88%, possibly due to the broad content coverage and overlap with other categories reflected in book covers.The classification outcomes reveal that certain categories with distinct features or consistent styles are more readily identifiable. However, for more generalised or diverse categories, achieving precise classification poses a challenge for the model, resulting in lower accuracy rates. Further research is warranted to enhance the model’s capability in classifying a broader range of book cover styles. Moreover, the accurate classification of specific categories, as highlighted in the table, offers a viable model solution for corresponding book management classification tasks.

**Table 5:** Accuracy values of 30 categories according to CPPDE-YOLO model classification.

Category	*Top_1 Accuracy/%*	*Top_5 Accuracy/%*
Arts & Photography	19.63	55.17
Biographies & Memoirs	26.83	66.40
Business & Money	17.11	62.03
Calendars	54.22	80.82
Children’s Books	39.72	73.24
Christian Books & Bibles	42.93	57.87
Comics & Graphic Novels	65.62	86.88
Computers & Technology	47.24	71.82
Cookbooks, Food & Wine	51.16	70.18
Crafts, Hobbies & Home	19.05	54.64
Engineering & Transportation	32.38	62.66
Health, Fitness & Dieting	7.20	44.88
History	33.16	66.06
Humour & Entertainment	10.96	51.96
Law	34.76	61.71
Literature & Fiction	17.38	53.56
Medical Books	25.77	60.97
Mystery, Thriller & Suspense	48.06	70.80
Parenting & Relationships	26.29	59.46
Politics & Social Sciences	9.39	57.11
Reference	19.01	48.44
Religion & Spirituality	19.48	56.73
Romance	55.07	75.12
Sciences & Math	21.57	61.42
Science & Fiction & Fantasy	36.27	66.91
Self-Help	20.94	59.78
Sports & Outdoors	15.75	53.54
Teen & Young Adult	14.84	47.80
Test Preparation	65.58	74.37
Travel	37.33	63.79
**All**	**30.7**	**62.7**

To mitigate bias from pre-training and ensure that the model learns from scratch, we opted not to load pre-training weights in the experiments. This decision facilitates a more rigorous assessment of the model structure’s capacity to learn and adapt to the dataset without relying on prior knowledge. It also aids in evaluating the performance of the model structure more accurately.

**Fig 12:**
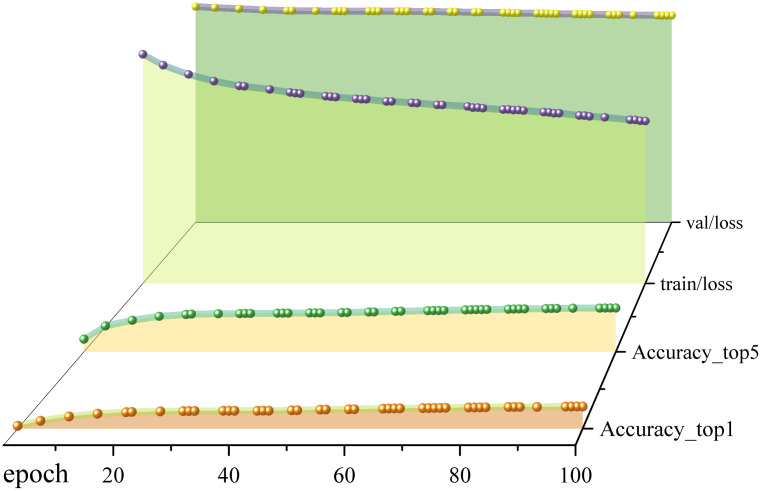
Accuracy and loss curves during the training of the CPPDE-YOLO model.

Comparing Table 6, we find that the *Top_1 Accuracy* and *Top_5 Accuracy* of the CPPDE-YOLO model on the dataset are higher than those of the YOLOv8 model by 1.1% and 1.0%, respectively. The *Top_1 Accuracy* and *Top_5 Accuracy* of the CPPDE-YOLO model are higher than those of the Ghost- YOLO model by 3.3% and 3.4%, respectively; the *Top_1 Accuracy* and *Top_5 Accuracy* of the CPPDE-YOLO model are 2.7% and 2.9% higher than those of the Faster-YOLO model in the dataset. Its trend is shown in Fig 12.This demonstrates the effectiveness and advantages of our proposed CPPDE-YOLO model in dealing with book cover feature acquisition and classification tasks. According to the information in the second part of the table, the *Top_1 Accuracy* of the CPPDE YOLO model is significantly higher than the non-YOLO architecture (16.1%−18.45%). This indicates that in the object classification task, the YOLO-based model has a significant robustness and advantage over other algorithmic systems in terms of multi-class discriminative ability, and that the CPPDE-YOLO model can effectively acquire global features and better utilise the features of the dataset.

**Table 6:** Comparison between the improved model and the original YOLOv8 classification model.

*Comparison of algorithms within the same series under identical dataset conditions (bookcover).*		
Algorithm Name	*Top_1 Accuracy/%*	*Top_5 Accuracy/%*
YOLOv8	29.6	61.7
Ghost-yolo	27.4	59.3
Faster-yolo	28.0	59.8
CPPDE-YOLO	**30.7**	**62.7**
***Classification accuracy results of different algorithm series under the same dataset conditions (bookcover).***		
**Algorithm Name**	***Top_1 Accuracy/%***	***Top_5 Accuracy/%***
Hawkeye	18.45	–
RepViT	16.1	22.3
CPPDE-YOLO	**30.7**	**62.7**

In order to comprehensively demonstrate the performance of the CPPDE-YOLO algorithm, experiments were conducted on cross-domain datasets. The Garbage Classification dataset (a publicly available dataset from the EXTREME MART platform) consists of images related to garbage classification, divided into a training set (85%) and a test set (15%). The Plants dataset contains 4503 images related to the health status of plants (taken from Kaggle).As shown in the Table 7, CPPDE-YOLO achieves a classification accuracy close to YOLOv8, indicating that both algorithms have similar discriminative capabilities on the primary categories within these datasets. However, CPPDE-YOLO offers significant time savings compared to YOLOv8, highlighting its improved robustness and effectiveness across different domains.

**Table 7:** Compare different datasets using the same algorithm.

Dataset	Algorithm Name	*Top_1 Accuracy*	*Top_5 Accuracy*
Garbage Classification	**CPPDE-YOLO**	**0.930**	**1**
	YOLOv8	0.928	1
Plants	**CPPDE-YOLO**	**0.927**	**1**
	YOLOv8	0.955	1

## Conclusion

We propose an enhanced image classification model, CPPDE-YOLO, based on YOLOv8, which aims to strike a balance between complex feature extraction and computational efficiency in book cover classification tasks. Experimental results on datasets show that the CPPDE-YOLO model achieves classification accuracy improvements of 1.1% and 1.0% over YOLOv8, respectively. These results confirm the effectiveness and advantages of our model in book cover classification, especially when dealing with complex global features.

Currently, the proliferation of book publications and the growing diversity of cover designs pose significant challenges to traditional classification methods in terms of accuracy and scalability. Through algorithmic innovation, CPPDE-YOLO provides an efficient automated solution for intelligent library management, using deep learning techniques to address the challenges of book classification characterised by complex features.

As deep learning algorithms are closely related to big data, our next goal is to integrate both textual and visual information from book covers for a more comprehensive management of book information. We will also explore the application of deep learning to other aspects of book management, such as e-books or cultural products such as films and videos. In addition, we aim to collect more experimental data to understand the correlations between book distribution, sales and local geographical information in order to build a more comprehensive and detailed modern book retrieval system.
